# Poly[[diaqua[μ_5_-(*R*,*S*)-2-({2-[(1,2-di­carboxyl­atoeth­yl)amino]­eth­yl}amino)­butane­dioato]cobaltate(III)sodium] di­hydrate]

**DOI:** 10.1107/S160053681104013X

**Published:** 2011-10-22

**Authors:** Olena K. Trunova, Anatolij V. Dudko, Tamara O. Makotryk, Olena V. Osadcha, Vasily I. Pekhnyo, Ganna V. Shovkova

**Affiliations:** a Institute of General and Inorganic Chemistry, NAS Ukraine, Kyiv, prosp. Palladina 32/34, 03680, Ukraine

## Abstract

In the asymmetric unit of the title coordination polymer, {[CoNa(C_10_H_12_N_2_O_8_)(H_2_O)_2_]·2H_2_O}_*n*_, the Co^II^ ion is coord­inated in a distorted octa­hedral environment, defined by two N atoms and four carboxyl­ate O atoms. Two Co^II^ ions and two 2-({2-[(1,2-dicarboxyl­atoeth­yl)amino]­eth­yl}amino)­butane­dio­ate (EDDS) ligands form a dimeric complex dianion [Co_2_(EDDS)_2_]. These dimeric units are connected *via* Na^+^ ions, forming a three-dimensional polymeric structure. In the crystal, the ligand N—H groups and the coordinated and solvent water mol­ecules are involved in inter­molecular N—H⋯O and O—H⋯O hydrogen bonding, reinforcing the three-dimensional polymeric structure.

## Related literature

For the synthesis and applications of EDDS and its complexes, see: Jones & Williams (2001 ▶); Kos & Leštan (2003 ▶); Mazurenko & Trunova (2001 ▶); Meers *et al.* (2005 ▶); Shadchina *et al.* (2008 ▶); Tandy *et al.* (2004 ▶, 2006 ▶); Vandevivere *et al.* (2001 ▶). For related structures, see: Horn *et al.* (1993 ▶); Pavelčík *et al.* (1980 ▶). For standard bond-length data, see: Allen *et al.* (1987 ▶). 
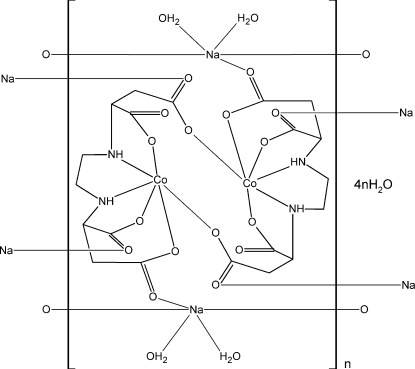


## Experimental

### 

#### Crystal data

[CoNa(C_10_H_12_N_2_O_8_)(H_2_O)_2_]·2H_2_O
*M* *_r_* = 442.20
Orthorhombic, 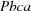

*a* = 10.0207 (2) Å
*b* = 15.6475 (2) Å
*c* = 20.3837 (4) Å
*V* = 3196.14 (10) Å^3^
*Z* = 8
Mo *K*α radiation
μ = 1.17 mm^−1^
*T* = 100 K
0.32 × 0.28 × 0.13 mm

#### Data collection

Bruker SMART APEXII diffractometer
Absorption correction: numerical (*SADABS*; Sheldrick, 1996 ▶) *T*_min_ = 0.706, *T*_max_ = 0.863
11219 measured reflections
3161 independent reflections
2549 reflections with *I* > 2σ(*I*)
*R*_int_ = 0.042

#### Refinement

*R*[*F* ^2^ > 2σ(*F* ^2^)] = 0.029
*wR*(*F* ^2^) = 0.065
*S* = 0.97
3161 reflections
265 parameters
1 restraint
H atoms treated by a mixture of independent and constrained refinement
Δρ_max_ = 0.34 e Å^−3^
Δρ_min_ = −0.50 e Å^−3^

### 

Data collection: *APEX2* (Bruker, 2007 ▶); cell refinement: *SAINT* (Bruker, 2007 ▶); data reduction: *SAINT*; program(s) used to solve structure: *SHELXTL* (Sheldrick, 2008 ▶); program(s) used to refine structure: *SHELXL97* (Sheldrick, 2008 ▶); molecular graphics: *DIAMOND* (Brandenburg & Putz, 2010 ▶); software used to prepare material for publication: *publCIF* (Westrip, 2010 ▶).

## Supplementary Material

Crystal structure: contains datablock(s) I, global. DOI: 10.1107/S160053681104013X/lh5329sup1.cif

Structure factors: contains datablock(s) I. DOI: 10.1107/S160053681104013X/lh5329Isup2.hkl

Additional supplementary materials:  crystallographic information; 3D view; checkCIF report

## Figures and Tables

**Table 1:** Hydrogen-bond geometry (Å, °)

*D*—H⋯*A*	*D*—H	H⋯*A*	*D*⋯*A*	*D*—H⋯*A*
N1—H1*N*⋯O7^i^	0.84 (2)	2.21 (2)	2.830 (2)	130.6 (18)
N2—H2*N*⋯O6^ii^	0.82 (2)	2.16 (2)	2.809 (2)	137.0 (18)
O9—H91⋯O11	0.84 (2)	1.92 (2)	2.741 (2)	166 (2)
O9—H92⋯O2^iii^	0.80 (3)	2.08 (3)	2.853 (2)	162 (3)
O10—H101⋯O8	0.83 (2)	2.18 (3)	2.972 (2)	160 (2)
O10—H102⋯O2^iv^	0.82 (3)	2.04 (3)	2.853 (2)	175 (3)
O11—H111⋯O1	0.79 (3)	2.26 (3)	3.004 (2)	158 (3)
O11—H112⋯O12^iii^	0.96 (3)	1.77 (3)	2.706 (3)	164 (2)
O12—H121⋯O1	0.77 (3)	2.05 (3)	2.793 (2)	161 (3)
O12—H122⋯O4^v^	0.76 (3)	2.04 (3)	2.788 (2)	167 (3)

Symmetry codes: (i) 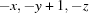; (ii) 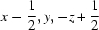; (iii) 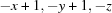; (iv) 
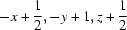
; (v) 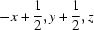.

**Fig. 1.**
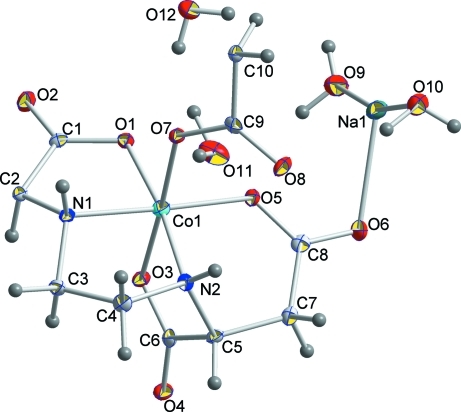
The asymmetric unit of the title compound with ellipsoids are drawn at the 50% probability level. H atoms are represented as small spheres of arbitrary radii.

**Fig. 2.**
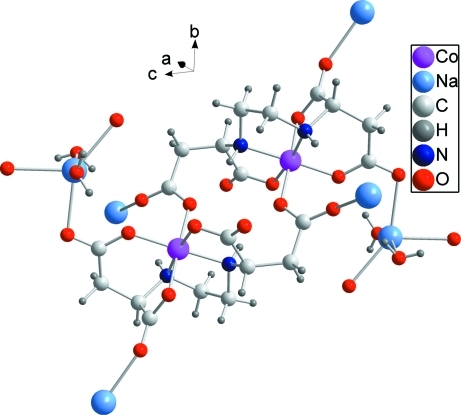
Part of the polymeric structure of the title compound formed by sodium ions.

**Fig. 3.**
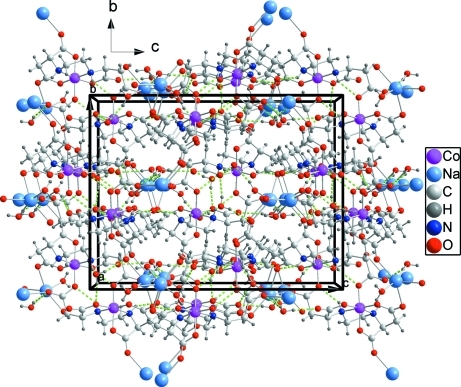
Crystal packing of title compound, projected along the a axis. Dashed lines indicate hydrogen bonds.

## supplementary crystallographic information

### Comment

Ethylenediamine-N,*N*'-disuccinic acid and its coordination compounds with different 3 d-metals have attracted much interest due to their potential and practical applications in biochemistry. EDDS can be applied for some technical purposes: for the extraction of heavy metals from soils as an efficient biodegradable chelating agents (Tandy *et al.*, 2004; Tandy *et al.*, 2006), in replacement of edta in soil washing and phytoextraction (Vandevivere *et al.*, 2001; Kos & Leštan, 2003; Meers *et al.* 2005) or for radionuclide decontamination in the pulp and paper-making industry (Jones & Williams, 2001). Biologically active complexes are widely used in plant growing and animal industries (Mazurenko & Trunova 2001; Shadchina *et al.* 2008). The biodegradable strong transition metal chelant [S,*S*] stereoisomer of ethylenediamine disuccinate was investigated for its applicability for the washing extraction of heavy metals from soil, sewage sludge, and harbor sediment (Vandevivere *et al.*, 2001).

In the course of our investigations the title compound  was prepared and structurally characterized. The asymmetric unit of the title compound is shown in Fig. 1. The Co^II^ ion forms a distorted octahedral [CoN_2_O_4_] environment defined by sets of three five-membered (Co1/O1/C1/C2/N1;/Co1/N1/C3/C4/N2; Co1/N2/C5/C6/O3) and a six-membered (Co1/N2/C5/C7/C8/O5) metallocycle. The sixth O donor from a centrosymmetrically related [Co(edds)]^-^ complex ion forms a 12-membered macrocycle, as a result a dimeric unit is produced (Fig. 2). The Co-O, Co-N, Na-O bond lengths are in normal ranges and have a good correlation with literature data (Allen *et al.*, 1987; Pavelčík *et al.*, 1980; Horn *et al.*, 1993). The Co1···Co1(-x, -y+1, -z) distance is 5.265 Å, which excludes the possibility of any interaction between the ions. In the crystal the compound exists as a polymeric structure, in which the monomers are interconnected by Na^+^ ions (Fig. 2). The Na^+^ ions are five-coordinate, with a distorted trigonal-bipyramidal coordination geometry formed by oxygen atoms, two of which belong to water molecules and the other three to oxygen atoms of the ligand which do not take part in the coordination of the Co^ii^ ion. Intermolecular N—H···O and O—H···O hydrogen bonds in the crystal structure form a three-dimensional supramolecular network which stabilizes the structure (Fig.3, Table 1).

### Experimental

A mixture of CoCl_2_.6H_2_O (2.38 g, 10 mmol) and EDDS (2.92 g, 10 mmol) were dissolved in distilled water (10 ml). The pH was then adjusted to 4.5 by concentrated NaOH solution. Reaction mixture was refluxed with stirring for 24 h. After cooling to room temperature diethyl ether was added into the solution giving a powder crude product. Precipitate was filtered of and washed with methanol for several times (yield 86%). The resulting residue was dissolved in water and was stored in a dark place for slow evaporation. After 4 days suitable crystals for X-ray data collection were obtained.

### Refinement

H atoms bonded to O and N atoms were located in a difference Fourier map. Their positions were refined freely whereas thermal parameters were fixed to *U*_iso_(H) = 1.5*U*_eq_(N,*O*). To avoid short contacts between H91 and H112 they were refined with distance restraint (H···H= 2.3 ±0.02 Å). H atoms bonded to C were positioned geometrically and refined using a riding model with C—H = 0.99 Å for CH_2_ and C—H = 1.00 Å for CH with *U*_iso_(H) = 1.2*U*_eq_(C).

### Crystal data

**Table:**

[CoNa(C_10_H_12_N_2_O_8_)(H_2_O)_2_]·2H_2_O	*F*(000) = 1824
*M**_r_* = 442.20	*D*_x_ = 1.838 Mg m^−^^3^
Orthorhombic, *P**b**c**a*	Mo *K*α radiation, λ = 0.71073 Å
Hall symbol: -P 2ac 2ab	Cell parameters from 4049 reflections
*a* = 10.0207 (2) Å	θ = 2.6–26.1°
*b* = 15.6475 (2) Å	µ = 1.17 mm^−^^1^
*c* = 20.3837 (4) Å	*T* = 100 K
*V* = 3196.14 (10) Å^3^	Diamond, violet
*Z* = 8	0.32 × 0.28 × 0.13 mm

### Data collection

**Table:**

Bruker SMART APEXII diffractometer	3161 independent reflections
Radiation source: fine-focus sealed tube	2549 reflections with *I* > 2σ(*I*)
graphite	*R*_int_ = 0.042
φ and ω scans	θ_max_ = 26.1°, θ_min_ = 2.0°
Absorption correction: numerical (*SADABS*; Sheldrick, 1996)	*h* = −7→12
*T*_min_ = 0.706, *T*_max_ = 0.863	*k* = −19→19
11219 measured reflections	*l* = −25→21

### Refinement

**Table:**

Refinement on *F*^2^	Primary atom site location: structure-invariant direct methods
Least-squares matrix: full	Secondary atom site location: difference Fourier map
*R*[*F*^2^ > 2σ(*F*^2^)] = 0.029	Hydrogen site location: inferred from neighbouring sites
*wR*(*F*^2^) = 0.065	H atoms treated by a mixture of independent and constrained refinement
*S* = 0.97	*w* = 1/[σ^2^(*F*_o_^2^) + (0.0324*P*)^2^] where *P* = (*F*_o_^2^ + 2*F*_c_^2^)/3
3161 reflections	(Δ/σ)_max_ < 0.001
265 parameters	Δρ_max_ = 0.34 e Å^−^^3^
1 restraint	Δρ_min_ = −0.50 e Å^−^^3^

### Special details

**Table:**

Geometry. All e.s.d.'s (except the e.s.d. in the dihedral angle between two l.s. planes) are estimated using the full covariance matrix. The cell e.s.d.'s are taken into account individually in the estimation of e.s.d.'s in distances, angles and torsion angles; correlations between e.s.d.'s in cell parameters are only used when they are defined by crystal symmetry. An approximate (isotropic) treatment of cell e.s.d.'s is used for estimating e.s.d.'s involving l.s. planes.
Refinement. Refinement of *F*^2^ against ALL reflections. The weighted *R*-factor *wR* and goodness of fit *S* are based on *F*^2^, conventional *R*-factors *R* are based on *F*, with *F* set to zero for negative *F*^2^. The threshold expression of *F*^2^ > σ(*F*^2^) is used only for calculating *R*-factors(gt) *etc*. and is not relevant to the choice of reflections for refinement. *R*-factors based on *F*^2^ are statistically about twice as large as those based on *F*, and *R*- factors based on ALL data will be even larger.

### Fractional atomic coordinates and isotropic or equivalent isotropic displacement parameters (Å^2^)

**Table:**

	*x*	*y*	*z*	*U*_iso_*/*U*_eq_	
Co1	0.09689 (3)	0.387117 (14)	0.083088 (13)	0.00826 (9)	
Na1	0.39217 (9)	0.53940 (5)	0.21505 (4)	0.0178 (2)	
C1	0.1871 (2)	0.38312 (11)	−0.04223 (10)	0.0104 (4)	
C2	0.0531 (2)	0.33752 (11)	−0.04526 (9)	0.0100 (4)	
H2	0.0712	0.2749	−0.0414	0.012*	
C3	−0.1234 (2)	0.29948 (11)	0.03843 (10)	0.0120 (5)	
H3A	−0.0830	0.2418	0.0400	0.014*	
H3B	−0.2034	0.2975	0.0099	0.014*	
C4	−0.1601 (2)	0.32957 (12)	0.10675 (10)	0.0130 (5)	
H4A	−0.2192	0.3801	0.1038	0.016*	
H4B	−0.2088	0.2838	0.1302	0.016*	
C5	0.0280 (2)	0.28136 (11)	0.18052 (10)	0.0111 (4)	
H5	−0.0412	0.2455	0.2027	0.013*	
C6	0.1054 (2)	0.22824 (12)	0.13122 (10)	0.0118 (4)	
C7	0.1248 (2)	0.31881 (11)	0.23126 (10)	0.0123 (5)	
H7A	0.0714	0.3483	0.2653	0.015*	
H7B	0.1714	0.2707	0.2529	0.015*	
C8	0.2302 (2)	0.38128 (11)	0.20641 (10)	0.0124 (5)	
C9	0.0287 (2)	0.55445 (11)	0.13005 (10)	0.0106 (4)	
C10	0.0175 (2)	0.64820 (11)	0.11074 (10)	0.0111 (4)	
H10A	0.1083	0.6729	0.1082	0.013*	
H10B	−0.0317	0.6792	0.1455	0.013*	
N1	−0.02558 (17)	0.36314 (9)	0.01328 (8)	0.0087 (4)	
H1N	−0.065 (2)	0.4093 (12)	0.0043 (10)	0.010*	
N2	−0.03629 (17)	0.35219 (10)	0.14370 (8)	0.0102 (4)	
H2N	−0.057 (2)	0.3904 (12)	0.1691 (11)	0.012*	
O1	0.22354 (14)	0.41008 (8)	0.01494 (6)	0.0105 (3)	
O2	0.25654 (16)	0.39053 (8)	−0.09163 (7)	0.0163 (3)	
O3	0.15026 (14)	0.27071 (7)	0.08149 (6)	0.0113 (3)	
O4	0.12621 (15)	0.15144 (8)	0.14003 (7)	0.0158 (3)	
O5	0.22710 (14)	0.41130 (8)	0.14795 (7)	0.0119 (3)	
O6	0.31703 (15)	0.40383 (8)	0.24573 (7)	0.0184 (4)	
O7	0.04285 (14)	0.50388 (7)	0.08043 (6)	0.0107 (3)	
O8	0.02390 (15)	0.53288 (8)	0.18835 (7)	0.0156 (3)	
O9	0.56412 (18)	0.49111 (10)	0.15130 (8)	0.0245 (4)	
H91	0.537 (3)	0.4471 (15)	0.1319 (11)	0.037*	
H92	0.605 (3)	0.5215 (16)	0.1272 (14)	0.037*	
O10	0.22692 (18)	0.62299 (10)	0.26905 (8)	0.0214 (4)	
H101	0.162 (3)	0.5949 (15)	0.2558 (12)	0.032*	
H102	0.229 (3)	0.6165 (15)	0.3088 (13)	0.032*	
O11	0.49078 (18)	0.36011 (9)	0.06961 (9)	0.0253 (4)	
H111	0.415 (3)	0.3726 (17)	0.0657 (14)	0.038*	
H112	0.539 (3)	0.3836 (15)	0.0329 (11)	0.038*	
O12	0.33610 (19)	0.57331 (10)	0.01853 (8)	0.0241 (4)	
H121	0.320 (3)	0.5253 (16)	0.0228 (13)	0.036*	
H122	0.348 (3)	0.5872 (17)	0.0539 (14)	0.036*	

### Atomic displacement parameters (Å^2^)

**Table:**

	*U*^11^	*U*^22^	*U*^33^	*U*^12^	*U*^13^	*U*^23^
Co1	0.00957 (17)	0.00764 (12)	0.00757 (14)	0.00002 (11)	−0.00016 (12)	0.00084 (11)
Na1	0.0234 (5)	0.0147 (4)	0.0153 (4)	0.0012 (4)	0.0001 (4)	0.0032 (3)
C1	0.0113 (12)	0.0080 (8)	0.0119 (10)	0.0028 (8)	−0.0009 (9)	0.0029 (8)
C2	0.0141 (12)	0.0078 (8)	0.0082 (10)	0.0000 (8)	0.0013 (9)	−0.0007 (8)
C3	0.0110 (12)	0.0123 (8)	0.0126 (11)	−0.0031 (8)	−0.0009 (9)	0.0017 (8)
C4	0.0081 (12)	0.0163 (9)	0.0145 (11)	−0.0004 (9)	−0.0002 (9)	0.0017 (8)
C5	0.0118 (12)	0.0109 (8)	0.0105 (10)	0.0002 (8)	−0.0009 (9)	0.0036 (8)
C6	0.0103 (12)	0.0137 (9)	0.0114 (10)	−0.0008 (9)	−0.0040 (9)	0.0008 (8)
C7	0.0142 (13)	0.0125 (9)	0.0103 (10)	0.0026 (9)	−0.0011 (9)	−0.0008 (8)
C8	0.0142 (13)	0.0097 (8)	0.0134 (11)	0.0043 (8)	−0.0005 (10)	0.0002 (8)
C9	0.0048 (12)	0.0128 (9)	0.0143 (11)	−0.0016 (8)	−0.0008 (9)	0.0000 (8)
C10	0.0135 (13)	0.0106 (9)	0.0092 (10)	−0.0004 (8)	0.0006 (9)	−0.0007 (8)
N1	0.0099 (10)	0.0072 (7)	0.0089 (9)	0.0014 (7)	−0.0001 (7)	0.0011 (7)
N2	0.0103 (10)	0.0099 (7)	0.0104 (9)	0.0017 (7)	−0.0001 (8)	−0.0010 (7)
O1	0.0091 (8)	0.0117 (6)	0.0107 (7)	−0.0014 (6)	0.0005 (6)	0.0008 (6)
O2	0.0159 (9)	0.0223 (7)	0.0107 (8)	−0.0029 (6)	0.0031 (7)	−0.0004 (6)
O3	0.0126 (8)	0.0099 (6)	0.0114 (7)	0.0018 (6)	0.0007 (6)	0.0014 (6)
O4	0.0241 (10)	0.0095 (6)	0.0138 (8)	0.0018 (6)	−0.0011 (7)	0.0015 (6)
O5	0.0116 (9)	0.0137 (6)	0.0103 (7)	−0.0014 (6)	−0.0021 (6)	0.0013 (6)
O6	0.0203 (9)	0.0198 (7)	0.0153 (8)	−0.0051 (6)	−0.0078 (7)	0.0024 (6)
O7	0.0138 (8)	0.0086 (6)	0.0097 (7)	0.0011 (6)	−0.0005 (6)	0.0008 (6)
O8	0.0229 (10)	0.0156 (6)	0.0083 (7)	0.0033 (6)	0.0017 (7)	0.0016 (6)
O9	0.0310 (12)	0.0211 (8)	0.0214 (9)	−0.0064 (7)	0.0051 (8)	−0.0005 (7)
O10	0.0232 (10)	0.0246 (8)	0.0163 (8)	−0.0030 (7)	−0.0002 (8)	0.0023 (7)
O11	0.0174 (10)	0.0180 (7)	0.0405 (11)	0.0026 (7)	0.0020 (9)	−0.0021 (7)
O12	0.0380 (12)	0.0140 (7)	0.0202 (9)	−0.0057 (7)	−0.0033 (9)	0.0003 (7)

### Geometric parameters (Å, °)

**Table:**

Co1—O5	1.8957 (14)	C5—C7	1.534 (3)
Co1—O3	1.8987 (12)	C5—H5	1.0000
Co1—N2	1.8990 (17)	C6—O4	1.233 (2)
Co1—O7	1.9064 (12)	C6—O3	1.293 (2)
Co1—O1	1.9155 (13)	C7—C8	1.526 (3)
Co1—N1	1.9161 (17)	C7—H7A	0.9900
Na1—O9	2.2866 (19)	C7—H7B	0.9900
Na1—O4^i^	2.3336 (15)	C8—O6	1.234 (2)
Na1—O6	2.3363 (15)	C8—O5	1.281 (2)
Na1—O8^ii^	2.3728 (16)	C9—O8	1.236 (2)
Na1—O10	2.3800 (19)	C9—O7	1.292 (2)
Na1—O5	2.9368 (15)	C9—C10	1.523 (2)
Na1—H101	2.60 (3)	C10—C2^iii^	1.527 (3)
C1—O2	1.229 (2)	C10—H10A	0.9900
C1—O1	1.292 (2)	C10—H10B	0.9900
C1—C2	1.522 (3)	N1—H1N	0.84 (2)
C2—N1	1.485 (2)	N2—H2N	0.82 (2)
C2—C10^iii^	1.527 (3)	O4—Na1^iv^	2.3336 (15)
C2—H2	1.0000	O8—Na1^v^	2.3728 (16)
C3—N1	1.489 (2)	O9—H91	0.84 (2)
C3—C4	1.515 (3)	O9—H92	0.80 (3)
C3—H3A	0.9900	O10—H101	0.83 (2)
C3—H3B	0.9900	O10—H102	0.82 (3)
C4—N2	1.494 (3)	O11—H111	0.79 (3)
C4—H4A	0.9900	O11—H112	0.96 (3)
C4—H4B	0.9900	O12—H121	0.77 (3)
C5—N2	1.486 (2)	O12—H122	0.76 (3)
C5—C6	1.517 (3)		
			
O5—Co1—O3	90.55 (6)	N2—C5—C6	107.20 (16)
O5—Co1—N2	95.02 (7)	N2—C5—C7	109.25 (15)
O3—Co1—N2	86.16 (6)	C6—C5—C7	109.43 (17)
O5—Co1—O7	91.37 (6)	N2—C5—H5	110.3
O3—Co1—O7	177.39 (6)	C6—C5—H5	110.3
N2—Co1—O7	95.43 (6)	C7—C5—H5	110.3
O5—Co1—O1	90.70 (6)	O4—C6—O3	123.80 (19)
O3—Co1—O1	88.91 (6)	O4—C6—C5	121.60 (18)
N2—Co1—O1	172.47 (6)	O3—C6—C5	114.56 (15)
O7—Co1—O1	89.30 (5)	C8—C7—C5	117.30 (17)
O5—Co1—N1	176.22 (7)	C8—C7—H7A	108.0
O3—Co1—N1	88.84 (6)	C5—C7—H7A	108.0
N2—Co1—N1	88.66 (7)	C8—C7—H7B	108.0
O7—Co1—N1	89.12 (6)	C5—C7—H7B	108.0
O1—Co1—N1	85.56 (7)	H7A—C7—H7B	107.2
O9—Na1—O4^i^	86.29 (6)	O6—C8—O5	121.08 (19)
O9—Na1—O6	95.44 (6)	O6—C8—C7	117.10 (18)
O4^i^—Na1—O6	146.32 (6)	O5—C8—C7	121.79 (18)
O9—Na1—O8^ii^	92.19 (6)	O8—C9—O7	126.13 (17)
O4^i^—Na1—O8^ii^	128.32 (6)	O8—C9—C10	120.57 (17)
O6—Na1—O8^ii^	85.30 (5)	O7—C9—C10	113.31 (16)
O9—Na1—O10	165.64 (6)	C9—C10—C2^iii^	113.63 (15)
O4^i^—Na1—O10	80.52 (6)	C9—C10—H10A	108.8
O6—Na1—O10	98.69 (6)	C2^iii^—C10—H10A	108.8
O8^ii^—Na1—O10	91.54 (6)	C9—C10—H10B	108.8
O9—Na1—O5	86.23 (5)	C2^iii^—C10—H10B	108.8
O4^i^—Na1—O5	99.39 (5)	H10A—C10—H10B	107.7
O6—Na1—O5	47.42 (5)	C2—N1—C3	116.47 (14)
O8^ii^—Na1—O5	132.10 (5)	C2—N1—Co1	108.02 (12)
O10—Na1—O5	101.46 (6)	C3—N1—Co1	107.29 (12)
O9—Na1—H101	163.8 (6)	C2—N1—H1N	107.7 (15)
O4^i^—Na1—H101	83.6 (5)	C3—N1—H1N	109.9 (14)
O6—Na1—H101	86.1 (5)	Co1—N1—H1N	107.0 (14)
O8^ii^—Na1—H101	104.0 (6)	C5—N2—C4	115.98 (15)
O10—Na1—H101	18.5 (5)	C5—N2—Co1	103.81 (12)
O5—Na1—H101	83.0 (6)	C4—N2—Co1	108.90 (13)
O2—C1—O1	123.23 (19)	C5—N2—H2N	109.7 (15)
O2—C1—C2	120.69 (18)	C4—N2—H2N	106.2 (16)
O1—C1—C2	116.04 (17)	Co1—N2—H2N	112.4 (15)
N1—C2—C1	108.02 (15)	C1—O1—Co1	113.93 (13)
N1—C2—C10^iii^	114.61 (16)	C6—O3—Co1	112.44 (12)
C1—C2—C10^iii^	112.07 (16)	C6—O4—Na1^iv^	144.52 (14)
N1—C2—H2	107.3	C8—O5—Co1	126.31 (13)
C1—C2—H2	107.3	C8—O5—Na1	78.66 (11)
C10^iii^—C2—H2	107.3	Co1—O5—Na1	148.08 (6)
N1—C3—C4	105.57 (15)	C8—O6—Na1	108.23 (12)
N1—C3—H3A	110.6	C9—O7—Co1	126.58 (12)
C4—C3—H3A	110.6	C9—O8—Na1^v^	143.85 (14)
N1—C3—H3B	110.6	Na1—O9—H91	107.0 (17)
C4—C3—H3B	110.6	Na1—O9—H92	122.9 (19)
H3A—C3—H3B	108.8	H91—O9—H92	111 (2)
N2—C4—C3	109.59 (17)	Na1—O10—H101	95.7 (18)
N2—C4—H4A	109.8	Na1—O10—H102	112.1 (18)
C3—C4—H4A	109.8	H101—O10—H102	106 (3)
N2—C4—H4B	109.8	H111—O11—H112	108 (3)
C3—C4—H4B	109.8	H121—O12—H122	102 (3)
H4A—C4—H4B	108.2		
			
O2—C1—C2—N1	−161.41 (17)	N1—Co1—O1—C1	−14.24 (13)
O1—C1—C2—N1	20.9 (2)	O4—C6—O3—Co1	179.19 (16)
O2—C1—C2—C10^iii^	−34.2 (2)	C5—C6—O3—Co1	−3.1 (2)
O1—C1—C2—C10^iii^	148.05 (16)	O5—Co1—O3—C6	76.97 (13)
N1—C3—C4—N2	−46.74 (19)	N2—Co1—O3—C6	−18.03 (14)
N2—C5—C6—O4	−152.14 (19)	O1—Co1—O3—C6	167.66 (13)
C7—C5—C6—O4	89.5 (2)	N1—Co1—O3—C6	−106.76 (14)
N2—C5—C6—O3	30.1 (2)	O3—C6—O4—Na1^iv^	169.60 (14)
C7—C5—C6—O3	−88.3 (2)	C5—C6—O4—Na1^iv^	−7.9 (4)
N2—C5—C7—C8	−53.7 (2)	O6—C8—O5—Co1	−177.17 (13)
C6—C5—C7—C8	63.4 (2)	C7—C8—O5—Co1	0.7 (3)
C5—C7—C8—O6	−172.07 (17)	O6—C8—O5—Na1	−19.22 (17)
C5—C7—C8—O5	10.0 (3)	C7—C8—O5—Na1	158.64 (18)
O8—C9—C10—C2^iii^	−146.6 (2)	O3—Co1—O5—C8	−64.38 (16)
O7—C9—C10—C2^iii^	33.4 (2)	N2—Co1—O5—C8	21.81 (16)
C1—C2—N1—C3	−150.78 (16)	O7—Co1—O5—C8	117.39 (15)
C10^iii^—C2—N1—C3	83.5 (2)	O1—Co1—O5—C8	−153.29 (15)
C1—C2—N1—Co1	−30.05 (16)	O3—Co1—O5—Na1	159.72 (13)
C10^iii^—C2—N1—Co1	−155.76 (12)	N2—Co1—O5—Na1	−114.09 (13)
C4—C3—N1—C2	164.59 (16)	O7—Co1—O5—Na1	−18.51 (13)
C4—C3—N1—Co1	43.47 (17)	O1—Co1—O5—Na1	70.80 (13)
O3—Co1—N1—C2	−64.06 (11)	O9—Na1—O5—C8	112.60 (12)
N2—Co1—N1—C2	−150.24 (12)	O4^i^—Na1—O5—C8	−161.79 (12)
O7—Co1—N1—C2	114.30 (11)	O6—Na1—O5—C8	11.67 (11)
O1—Co1—N1—C2	24.94 (11)	O8^ii^—Na1—O5—C8	23.07 (14)
O3—Co1—N1—C3	62.25 (12)	O10—Na1—O5—C8	−79.65 (12)
N2—Co1—N1—C3	−23.93 (12)	O9—Na1—O5—Co1	−102.28 (13)
O7—Co1—N1—C3	−119.39 (12)	O4^i^—Na1—O5—Co1	−16.67 (14)
O1—Co1—N1—C3	151.24 (12)	O6—Na1—O5—Co1	156.79 (15)
C6—C5—N2—C4	78.7 (2)	O8^ii^—Na1—O5—Co1	168.18 (11)
C7—C5—N2—C4	−162.84 (17)	O10—Na1—O5—Co1	65.47 (14)
C6—C5—N2—Co1	−40.72 (17)	O5—C8—O6—Na1	25.3 (2)
C7—C5—N2—Co1	77.76 (16)	C7—C8—O6—Na1	−152.67 (13)
C3—C4—N2—C5	−88.7 (2)	O9—Na1—O6—C8	−92.30 (14)
C3—C4—N2—Co1	27.91 (17)	O4^i^—Na1—O6—C8	−0.8 (2)
O5—Co1—N2—C5	−57.29 (12)	O8^ii^—Na1—O6—C8	175.94 (14)
O3—Co1—N2—C5	32.93 (12)	O10—Na1—O6—C8	85.10 (14)
O7—Co1—N2—C5	−149.15 (12)	O5—Na1—O6—C8	−12.52 (12)
N1—Co1—N2—C5	121.86 (12)	O8—C9—O7—Co1	−14.0 (3)
O5—Co1—N2—C4	178.59 (11)	C10—C9—O7—Co1	165.99 (13)
O3—Co1—N2—C4	−91.20 (12)	O5—Co1—O7—C9	−40.90 (16)
O7—Co1—N2—C4	86.72 (12)	N2—Co1—O7—C9	54.28 (17)
O2—C1—O1—Co1	−178.47 (14)	O1—Co1—O7—C9	−131.58 (16)
C2—C1—O1—Co1	−0.80 (19)	N1—Co1—O7—C9	142.85 (17)
O5—Co1—O1—C1	165.23 (12)	O7—C9—O8—Na1^v^	−123.2 (2)
O3—Co1—O1—C1	74.69 (13)	C10—C9—O8—Na1^v^	56.8 (3)
O7—Co1—O1—C1	−103.41 (13)		

Symmetry codes: (i) −*x*+1/2, *y*+1/2, *z*; (ii) *x*+1/2, *y*, −*z*+1/2; (iii) −*x*, −*y*+1, −*z*; (iv) −*x*+1/2, *y*−1/2, *z*; (v) *x*−1/2, *y*, −*z*+1/2.

### Hydrogen-bond geometry (Å, °)

**Table:**

*D*—H···*A*	*D*—H	H···*A*	*D*···*A*	*D*—H···*A*
N1—H1N···O7^iii^	0.84 (2)	2.21 (2)	2.830 (2)	130.6 (18)
N2—H2N···O6^v^	0.82 (2)	2.16 (2)	2.809 (2)	137.0 (18)
O9—H91···O11	0.84 (2)	1.92 (2)	2.741 (2)	166 (2)
O9—H92···O2^vi^	0.80 (3)	2.08 (3)	2.853 (2)	162 (3)
O10—H101···O8	0.83 (2)	2.18 (3)	2.972 (2)	160 (2)
O10—H102···O2^vii^	0.82 (3)	2.04 (3)	2.853 (2)	175 (3)
O11—H111···O1	0.79 (3)	2.26 (3)	3.004 (2)	158 (3)
O11—H112···O12^vi^	0.96 (3)	1.77 (3)	2.706 (3)	164 (2)
O12—H121···O1	0.77 (3)	2.05 (3)	2.793 (2)	161 (3)
O12—H122···O4^i^	0.76 (3)	2.04 (3)	2.788 (2)	167 (3)

Symmetry codes: (iii) −*x*, −*y*+1, −*z*; (v) *x*−1/2, *y*, −*z*+1/2; (vi) −*x*+1, −*y*+1, −*z*; (vii) −*x*+1/2, −*y*+1, *z*+1/2; (i) −*x*+1/2, *y*+1/2, *z*.
